# Investigation of Charging Efficiency of a Lithium-ion Capacitor during Galvanostatic Charging Method

**DOI:** 10.3390/ma12193191

**Published:** 2019-09-29

**Authors:** Shunji Nakata

**Affiliations:** Department of Electronic Engineering and Computer Science, Faculty of Engineering, Kindai University, Takaya, Higashi-Hiroshima 739-2116, Japan; nakata@hiro.kindai.ac.jp

**Keywords:** charging efficiency, lithium-ion capacitor, adiabatic charging, energy dissipation

## Abstract

The charging efficiency of a lithium-ion capacitor (LIC) is an important problem. Until now, due to the stepwise charging method, the charging efficiency of 95.5% has been realized. However, the problem is that the issue of what level the charging efficiency can be increased to, is yet to be well investigated. In this article, the problem is investigated under the galvanostatic charging condition. The charging efficiency is measured as a function of the charging current. As a result, it can be more than 99.5% when the charging is quasi-static, in other words, an adiabatic process is realized. Next, the problem of how much energy can be taken out from the energy-stored capacitor is investigated with a load resistor circuit. It is clarified that the discharging energy from the capacitor is equal to the stored energy in the case when a load resistor is used and the discharging is quasi-static. It is confirmed that LICs are suitable for use as energy storage devices.

## 1. Introduction

Recently, the energy storage of renewable energy is an important problem. To store energy, supercapacitors are considered to play an important role. In particular, lithium-ion capacitors (LICs) have attractive characteristics [1,2,3,4,5,6]. The LICs have many advantages over lithium-ion batteries. The power density is 10 times larger and the operating temperature has a wider range [1]. The cycle lifetime of LICs is 1,000,000 cycles [2], while that of lithium-ion batteries is about 2000 cycles [7,8]. Another important point is that LICs are much safer than lithium-ion batteries. On the other hand, there is a drawback, such as energy density. The energy density of commercial LICs is 13 Wh/kg [9], while that of lithium-ion batteries is 100 Wh/kg [1], i.e., LICs are 13% that of the batteries. However, when there is a large space, it is not a serious problem. These advantageous characteristics make LICs very suitable for storing energy from renewable resources.

Dissipationless adiabatic charging of a capacitor has been researched [10,11,12,13,14,15,16,17,18,19,20,21,22,23,24,25,26,27,28,29,30]. Recently, a stepwise charging and discharging circuit has been proposed, which does not need a shunt resistor for detecting the current [23,26,27,29]. The proposed circuit is composed of switching transistors and an inductor. By changing the duty ratio of transistors digitally, stepwise charging is realized, which in turn, means the realization of adiabatic charging. The digital control of duty ratio is achieved by a microprocessor (PIC16F627A) (Microchip Technology, Chandler, AZ, USA), which outputs the Pulse Width Modulation (PWM) signal [27,29]. It was clarified experimentally that the charging efficiency is 95.5% during charging and also discharging. However, the question remains—to what level can the charging efficiency be increased? In this article, the charging efficiency of the LIC is investigated with the constant current or galvanostatic charging method and investigated as a function of the charging current. The paper is organized as follows: Section 2 gives the charging measurement system and experimental results, Section 3 presents the discharging measurement system from the energy-stored capacitor and the experimental results, Section 4 discusses the experimental results. The conclusion is given in Section 5.

## 2. Charging Experiment

**Figure 1:**
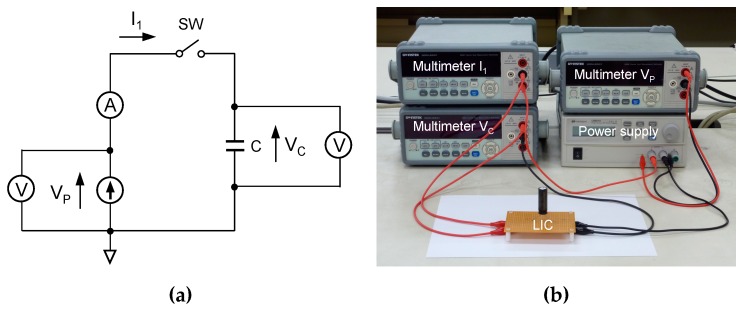
(**a**) Measurement system used when galvanostatic charging is performed. *I*_1_ is the charging current from the power supply to the lithium-ion capacitator (LIC); (**b**) photograph of the measurement system.

Figure 1 shows the measurement system in this experiment. The power supply is Agilent Technologies U8001A (Agilent Technologies, Inc., Santa Clara, CA, USA). Three multimeters are used for measuring the output voltage of power supply *V_P_*, the current flowing from the power supply into the capacitor *I*_1_, and the capacitor voltage *V_C_*. To realize galvanostatic charging, the maximum output current is set on U8001A. The LIC in this experiment is LIC1235RS3R8406 (Taiyo Yuden Co., Ltd., Tokyo, Japan). The capacitance is 40 F. The rated current of the capacitor is 2 A. The energy density of the LIC is 10 Wh/kg. The minimum and maximum operating voltages are 2.2 and 3.8 V. In this experiment, for the sufficient voltage margin from the voltage limit, the initial and final capacitor voltages during charging are set to 2.38 and 3.60 V, respectively. In the measurement, the timing of data capture is from the beginning, 2.38 V, to the ending point, 3.6 V.

**Figure 2:**
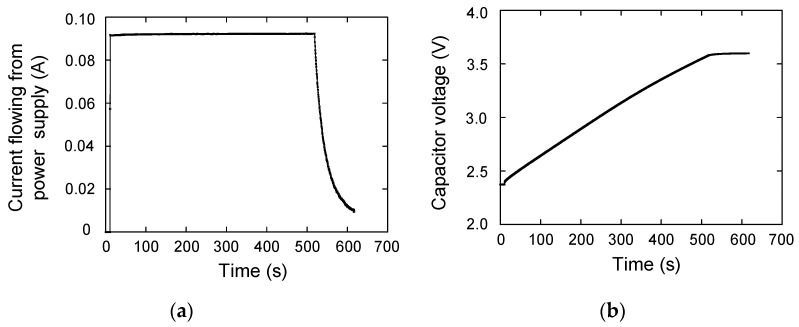
(**a**) Capacitor charging current as a function of time; (**b**) capacitor voltage as a function of time.

**Figure 3:**
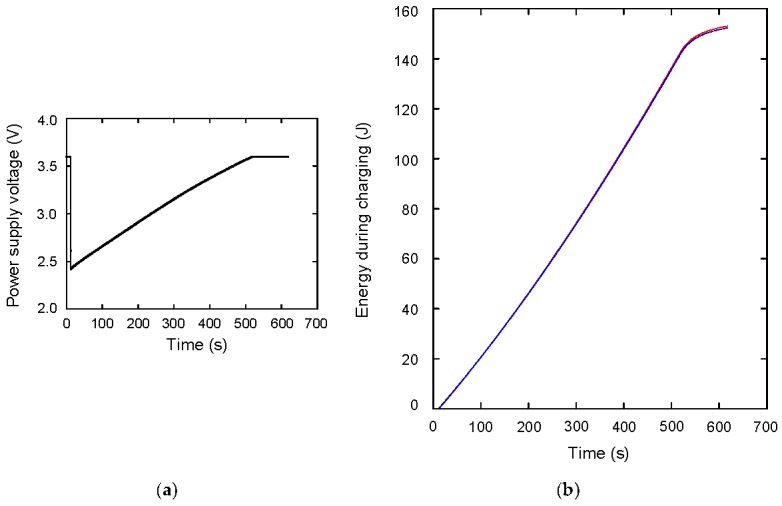
(**a**) Power supply output voltage as a function of time; (**b**) work done by the power supply (red line) and stored energy of the capacitor (blue line).

First, the maximum voltage and the maximum charging current are set to 3.6 V and 0.09 A on the power supply, respectively. Figure 2a shows *I*_1_. When *t* = 11 s, the power supply and the capacitor are connected. The current is increased from 0 to 0.09 A. When *t* = 520s, the capacitor voltage is close to 3.6 V. Then, the power supply becomes a constant voltage charging mode, and the current decreases rapidly from 0.09 A. The *V_C_* is shown in Figure 2b. The value reaches 3.60 V at *t* = 620 s, finally. Figure 3a shows *V_P_*. In the beginning, the value is 3.6 V and is decreased rapidly to 2.4 V at *t* = 11 s after the power supply and capacitor are connected. It then reaches to 3.60 V at *t* = 520 s.

Here, the C-rate of the charging current is considered. The capacity of the LIC is calculated to be 40 F × (3.8 V−2.2 V) = 64 C. Therefore, a 1 C-rate is 64 C/3600 s = 0.0178 A. Then, 0.09 A is equal to a 5.1 C-rate.

**Figure 4:**
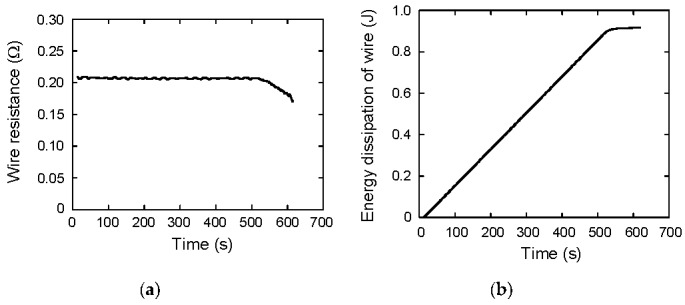
(**a**) Wire resistance as a function of time; (**b**) energy dissipation of the wire as a function of time.

From these measurement results, the work done by the power supply *E_P_* is calculated as *E_P_* = ∫*I*_1_*V*_P_ d*t*. On the other hand, the stored energy in the capacitor during charging *E_C_*_1_ is calculated as *E_C_*_1_ = ∫*I*_1_*V*_C_ d*t*. The suffix 1 means it is related to the charging, and the suffix 2 is used for the discharging process, which is discussed in the next section. The *E_P_* and *E_C_*_1_ are shown in Figure 3b as the red and blue lines, respectively. These are almost consistent, which means the charging efficiency *η* is almost 1. At *t* = 620 s, *E_P_* and *E_C_*_1_ are 153.11 and 152.33 J, respectively, so *η* is calculated to be *E_C_*_1_/*E_P_* = 0.995. The energy loss is considered to be the energy dissipation in wire resistance. This wire resistance is calculated from the experiment, and also the energy dissipation is calculated. Figure 4a shows the wire resistance *R_W_* as a function of time, which is calculated from (*V_P_*−*V_C_*)/*I*_1_. The average value during galvanostatic charging is 0.21 Ω. The energy dissipation can be calculated by *E_diss_* = ∫*R_W_ I*_1_^2^ d*t*, and it is shown in Figure 4b. The value reaches to 0.92 J at *t* = 620 s, which is almost consistent with the difference between *E_P_* and *E_C_*_1_, 0.78 J.

**Figure 5:**
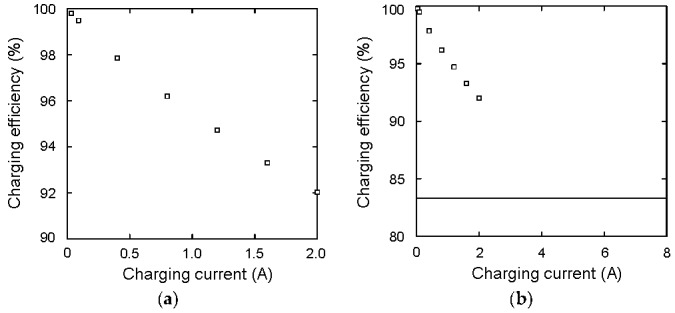
(**a**) Charging efficiency as a function of current; (**b**) comparison between galvanostatic charging and constant voltage charging (solid line).

Figure 5a shows the charging efficiency as a function of the charging current. The charging current values are 0.03, 0.09, 0.4, 0.8, 1.2, 1.6, and 2.0 A. The rated current is 2.0 A in this capacitor, so the experiment over 2.0 A was not performed. These currents correspond to a 1.7, 5.1, 22.5, 45.0, 67.5, 90.0, and 112.5 C-rate, respectively. The charging efficiencies to those current values are 99.8%, 99.5%, 97.9%, 96.2%, 94.7%, 93.3%, and 92.0%, respectively. For a profound understanding, the charging efficiency during constant voltage charging is considered. In the experiment, *V_C_* is changed from 2.4 to 3.6 V. Therefore, for easy calculation, the initial and final voltages are set to 2*V*/3 and *V*, respectively, where *V* is the constant power supply voltage. The charge amount difference Δ*Q* is *CV*/3, where *C* is the capacitance. So the work done by the power supply *W* is *W* = Δ*Q*×*V* = *CV*^2^/3. On the other hand, electrostatic energy difference during charging Δ*E*_1_ is written as Δ*E*_1_ = *C*/2×{*V*^2^ − (2*V*/3)^2^} = 5/18·*CV*^2^. Therefore, the charging efficiency is calculated to be *η* = Δ*E*_1_/*W* = 83.3%, which is shown in Figure 5b as a solid line. For comparison, the previous charging efficiencies are also plotted. If the galvanostatic charging current increased over 2 A, the charging efficiency would approach to 83.3%.

## 3. Discharging Experiment

**Figure 6:**
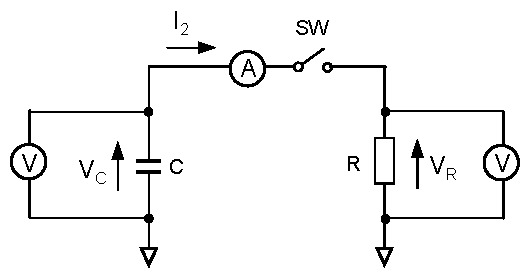
Measurement system when discharging is performed. *I*_2_ is the discharging current from the energy-stored capacitor to a load resistor.

**Figure 7:**
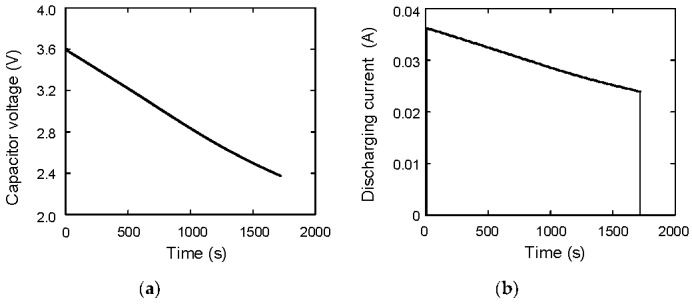
(**a**) Capacitor voltage as a function of time during discharging; (**b**) discharging current as a function of time.

**Figure 8:**
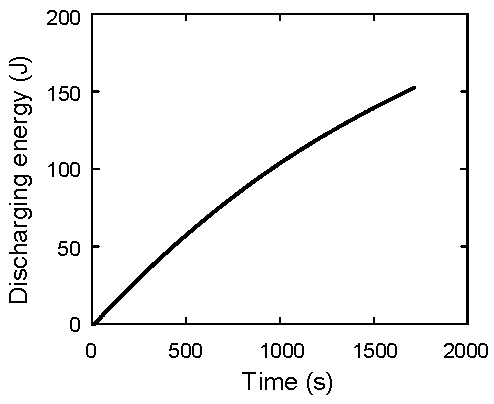
Discharging energy taken out from a capacitor.

In the previous section, the stored energy during charging is discussed. Here, the problem of how much energy can be taken out from the energy-stored capacitor is discussed. The discharging experimental system is shown in Figure 6. The discharging current flows from the capacitor to a load resistor. The capacitor voltage, the current flowing from the capacitor *I*_2_, and the load resistor voltage *V_R_* are measured with three multimeters. The nominal load resistance is 100 Ω. The *V_C_* during discharging is shown in Figure 7a. The initial and final voltages are 3.60 and 2.38 V, respectively. After 1720 s, *V_C_* reaches to 2.38 V, which is the same as the initial voltage when charging is performed. The *I*_2_ is shown in Figure 7b. When *t* = 0, a switch is open and current does not flow. Therefore, *I*_2_ is zero. When *t* = 10 s, a switch is closed and current flows. Then, *I*_2_ increases rapidly to 0.036 A. When *t* = 1720 s, the switch is open and *I*_2_ becomes 0 A. Here, the discharging energy *E_C_*_2_ is calculated as *E_C_*_2_ = ∫*I*_2_*V_C_* d*t*. The calculated result is shown in Figure 8. When *t* = 1720 s, *E_C_*_2_ becomes 152.62 J, which is consistent with the value of *E_C_*_1_, 152.33 J, and the difference between them is only 0.19%.

**Figure 9:**
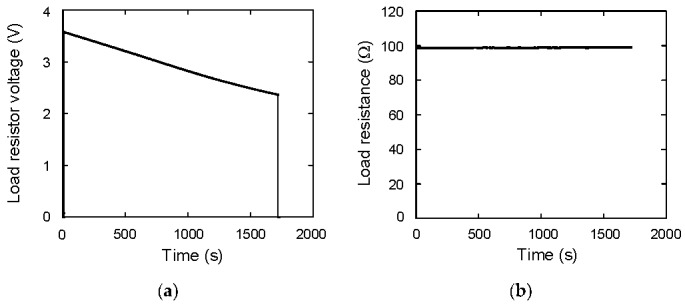
(**a**) Load resistor voltage as a function of time; (**b**) load resistance as a function of time.

Next, the discharging energy is investigated from the viewpoint of the Joule heat of the resistor in the circuit, which is composed of the load resistor and the wire one. The actual load resistance *R_L_* is calculated as *V_R_*/*I*_2_. The *V_R_* during discharging is shown in Figure 9a. When *t* = 0, a switch is open and current does not flow. Therefore, *V_R_* is zero. When *t* = 10 s, current flows and *V_R_* increases to 3.6 V, which is almost consistent with *V_C_*. When *t* = 1720 s, a switch is open and current does not flow. Then, *V_R_* becomes 0 V. Using *V_R_*, *V_R_*/*I*_2_ is calculated, which is shown in Figure 9b. The average value is 98.8 Ω, which is consistent with the nominal value.

**Figure 10:**
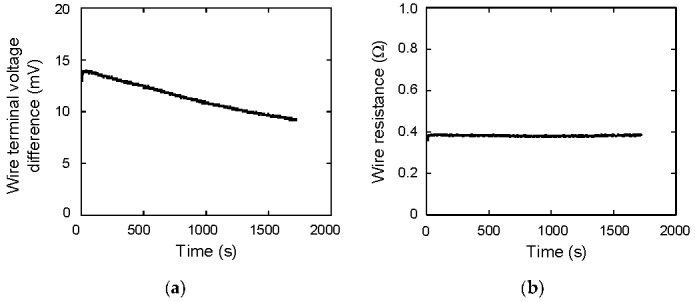
(**a**) Wire terminal voltage difference as a function of time; (**b**) wire resistance as a function of time.

**Figure 11:**
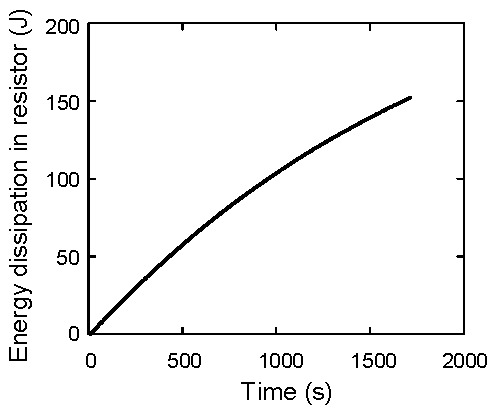
Energy dissipation caused at the resistor.

Using the wire terminal voltage difference *V_C_*−*V_R_*, the wire resistance *R_W_* is calculated as (*V_C_*−*V_R_*)/*I*_2_. The value of *V_C_*−*V_R_* is shown in Figure 10a. The calculated value of *R_W_* is shown in Figure 10b. The average value is 0.383 Ω. Then, the energy dissipation due to the Joule heat is calculated as *E_diss_* = ∫(*R_L_*+*R_W_*) *I*_2_^2^ d*t*. The result is shown in Figure 11. At *t* = 1720 s, *E_diss_* reaches to 152.41 J. This value is consistent with the previously calculated value of *E_C_*_1_ and *E_C_*_2_.

## 4. Discussion

In the energy storage device, the coulombic efficiency is discussed, which is defined as *C_d_*/*C_c_*, where *C_d_* is the discharging capacity and *C_c_* is the charging capacity [31]. In the voltage range from 2.38 to 3.6 V in this experiment, *C_c_* and *C_d_* are almost the same and they are 50.46 and 50.45 C, respectively. The coulombic efficiency is calculated to be 0.9996.

Here, the Peukert effect is considered. It is written as *C_n_*_1_ = *C_n_*(*I_n_*/*I_n_*_1_)*^k^*^−1^, where *I_n_* is the manufacturer-specified discharge current, *C_n_* is the rated capacity at *I_n_*, *I_n_*_1_ is a different discharge current, *C_n_*_1_ is the available capacity at *I_n_*_1_, and *k* is the Peukert coefficient [32]. From this equation, when *I_n_*_1_ becomes large, *C_n_*_1_ becomes small. This equation is applied not only to lead-acid and Li-ion batteries but also to Electric Double-Layer Capacitors (EDLCs) [33] and LICs [34,35,36] in the wide current range.

Then, the case where *I*_2_ becomes large is considered. The *E_C_*_2_ is rewritten as follows: *E_C_*_2_ = ∫*I*_2_*V_C_*d*t* = *I*_2_∫*V_C_*d*t* = *V_av_I*_2_*T*, where *T* is the discharging time and *V_av_* is the average voltage defined as ∫*V_C_*d*t*/*T*. If *I*_2_ is large, the capacity *I*_2_*T* becomes small due to the Peukert effect. Therefore, *E_C_*_2_ becomes small, which means the total efficiency of charging and discharging *E_C_*_2_/*E_P_* decreases. For increasing the total efficiency, the discharging current should be small, in other words, an adiabatic process has to be performed.

Regarding the energy efficiency of LICs, there are the following literature references. Reference [37] describes the efficiency of the LIC-based distributed uninterruptible power supply (UPS system. It shows that the experimental efficiency of the LIC with 3300 F is about 95% when the LIC module voltage changes from 6 to 7 V.

Reference [38] describes the efficiency of LICs (JM Energy) is 97% from simulation results. Reference [39] shows the efficiency of LICs is 80% from experimental results.

In this experiment, it is clarified that the charging efficiency changes from 92% to 99.8% systematically as a function of galvanostatic current.

## 5. Conclusions

For energy storage of renewable resources, LICs are attractive for the lifetime and safety in comparison with lithium-ion batteries. Regarding the energy density, the commercial LICs have a large energy density (13 Wh/kg) [9], which is close and comparable to that of lead-acid batteries. Therefore, LICs are promising for energy storage. Until now, the charging efficiency of the LIC was investigated with a stepwise charging circuit, and it was 95.5%. In this article, this charging efficiency is investigated in detail with the galvanostatic method. As a result, in the adiabatic charging, the charging efficiency is more than 99.5%. The energy dissipation is due to the Joule heat of the wire resistance. Next, how much energy in charging can be taken out during discharging was investigated. The experiment shows the discharging energy is equal to the energy during charging. This is due to no Joule heat energy dissipation for the adiabatic process and no leakage current for the LIC characteristic. From the result, the LIC causes no energy dissipation in the adiabatic process, and it is useful for energy storage devices.
